# The emergence of the tetrathionate reductase operon in the *Escherichia coli/Shigella* pan‐genome

**DOI:** 10.1002/mbo3.1333

**Published:** 2022-11-06

**Authors:** Floyd G. Adsit, Thomas A. Randall, Jacqueline Locklear, David M. Kurtz

**Affiliations:** ^1^ Quality Assurance Laboratory (QAL), Comparative Medicine Branch (CMB) National Institute of Environmental Health Sciences (NIEHS) Durham North Carolina USA; ^2^ Integrative Bioinformatics National Institute of Environmental Health Sciences (NIEHS) Durham North Carolina USA

**Keywords:** *Escherichia coli*, pathogen, *Shigella*, tetrathionate respiration, virulence factor

## Abstract

*Escherichia coli* pathogenic variants (pathovars) are generally characterized by defined virulence traits and are susceptible to the evolution of hybridized identities due to the considerable plasticity of the *E. coli* genome. We have isolated a strain from a purified diet intended for research animals that further demonstrates the ability of *E. coli* to acquire novel genetic elements leading potentially to emergent new pathovars. Utilizing next generation sequencing to obtain a whole genome profile, we report an atypical strain of *E. coli*, EcoFA807‐17, possessing a tetrathionate reductase (*ttr*) operon, which enables the utilization of tetrathionate as an electron acceptor, thus facilitating respiration in anaerobic environments such as the mammalian gut. The *ttr* operon is a potent virulence factor for several enteric pathogens, most prominently *Salmonella enterica*. However, the presence of chromosomally integrated tetrathionate reductase genes does not appear to have been previously reported in wild‐type *E. coli* or *Shigella*. Accordingly, it is possible that the appearance of this virulence factor may signal the evolution of new mechanisms of pathogenicity in *E. coli* and *Shigella* and may potentially alter the effectiveness of existing assays using tetrathionate reductase as a unique marker for the detection of *Salmonella enterica*.

## Graphical abstract

In recent years, the severity of some outbreaks of food‐borne illness ascribed to *Escherichia coli* has been recognized as due to enhanced virulence brought about by altered genetic characteristics of the implicated pathovars. Herein, we report the isolation of a strain of *E. coli* possessing a virulence factor normally associated with gastrointestinal infections by Salmonella or Citrobacter. We further provide a detailed examination of the extent and distribution of the tetrathionate reductase genes within strains of *E. coli*, *Shigella flexneri*, and *Shigella sonnei* deposited in Genbank.

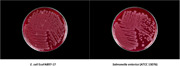

## 1 INTRODUCTION

Certain prokaryotes are capable of metabolizing dietary sulfur into hydrogen sulfide (H_2_S) which can be toxic to mammalian hosts (Nguyen et al., 2020). As part of a remedial mechanism for the detoxification of hydrogen sulfide, the mammalian cecal mucosa converts H_2_S to thiosulfate (Furne et al., 2001). Subsequently, thiosulfate can then be oxidized by these bacteria to tetrathionate (S_4_O_6_²¯) (Barton et al., 2017).

The ability to conduct anaerobic respiration using tetrathionate as an electron acceptor by members of the Enterobacteriaceae family is most commonly found in the genera *Salmonella, Proteus*, and *Citrobacter* (Barrett & Clark, 1987). These genera are extensively populated with obligate or opportunistic gastrointestinal pathogens. Their close relative, the species *Escherichia coli*, is a well‐characterized fecal coliform usually acting as a commensal but is also capable of causing intestinal and/or extraintestinal disease. Intestinal pathogenic *E. coli* strains can be divided into six separate categories or pathotypes including enteroaggregative *E. coli* (EAEC), entero‐invasive *E. coli* (EIEC), enteropathogenic *E. coli* (EPEC), enterotoxigenic *E. coli* (ETEC), enterohaemorrhagic *E. coli* (EHEC) and diffuse adhering *E. coli* (DAEC) (Chaudhuri et al., 2010). These *E. coli* pathovars share a core genome of approximately 2,000 genes. In the continuing evolution of *E. coli*, the core genomes are augmented by the nonhereditary acquisition of different genes of introgressive origin (Bapteste et al., 2012). As a consequence of the genomic acquisition, a variety of strains emerge possessing an array of adaptive or pathogenic traits (Baquero & Tobes, 2013), thereby displaying a remarkable genomic fluidity (Brunder & Karch, 2000; Hazen et al., 2017; Pasqua et al., 2017; Prager et al., 2014). Perhaps a rather dramatic example of this is the major outbreak caused by *Escherichia coli* of serotype O104:H4 which spread throughout Europe in 2011. This event, regarded as the most lethal of its kind ever reported (Sloup et al., 2016), was caused by an atypical strain that is most similar to enteroaggregative *E. coli* (EAEC) of serotype O104:H4. This EAEC variant, however, was found to possess a prophage encoding the Shiga toxin, which is characteristic of enterohemorrhagic *E. coli* (EHEC) strains. This combination of genomic features, uniting virulence factors from both EAEC and EHEC, represents an example of an emergent new pathovar (Navarro‐Garcia, 2014) designated as Enteroaggregative Enterohemorrhagic *E. coli*, or EAHEC. Introgressive descent refers to “the incorporation (usually via hybridization and backcrossing) of alleles from one entity (species) into the gene pool of a second, divergent entity (species)” (Harrison & Larson, 2014). In this report, we demonstrate a further example of this concept with the discovery of a strain possessing genetic elements previously unreported in *E. coli*.

**Figure 1:**
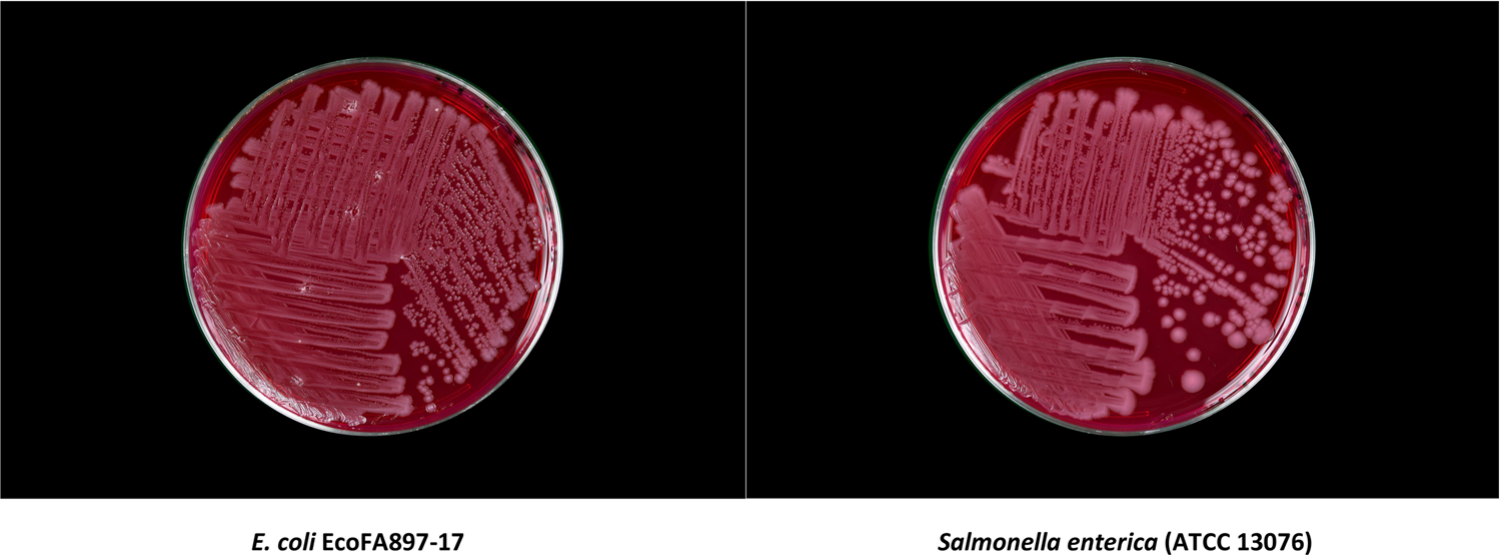
*Escherichia coli* EcoFA807‐17 and *Salmonella enterica* (96‐hour cultures) on brilliant green (BG) agar.

Our laboratory routinely tests research animal feeds used at our institute for microbial burden and food‐borne pathogens such as *Salmonella*. While screening a purified, high‐fat rodent diet, we isolated a novel strain of *E. coli* designated EcoFA807‐17, displaying an indeterminate biochemical profile while conducting an enrichment protocol for the detection of *Salmonella enterica*. *S*. *enterica* shares a morphological resemblance with EcoFA807‐17 on Brilliant Green (Figure 1) and MacConkey Agars, which is also remarkable in the case of Brilliant Green because this selective plate medium tends to inhibit the culture of many strains of *E. coli (*Moats & Kinner, 1974 *)* including Shigellae (Kristensen, 1925; Moats & Kinner, 1974). After initially characterizing the isolate via multi‐locus sequence analysis (MLSA) as *E. coli* rather than *Salmonella*, and upon completion of whole genome sequencing (WGS), the isolate appears to possess certain known virulence genes and phenotypes suggesting pathogenic potential, including a functional tetrathionate reductase operon. Tetrathionate respiration, conducted under conditions of anaerobiosis and gut inflammation, serves to enable *ttr*(+) prokaryotes to outcompete the endogenous intestinal microbiome and is therefore considered a virulence marker in certain enteric pathogens, most notably *Salmonella enterica* (Winter & Bäumler, 2011).

## 2 METHODS AND MATERIALS

### 2.1 Isolation and morphologic/biochemical phenotyping of EcoFA807‐17

EcoFA807‐17 was initially isolated from culture derived from Rappaport‐Vassiliadis broth, then subcultured upon MacConkey (MAC) and Brilliant Green (BG) agars. To initially confirm/disprove *Salmonella* with which the isolate shared a morphological resemblance on both of these two differential agars, cultures were grown on Triple Sugar Iron (TSI) slant media testing for H_2_S production (Binet et al., 2018). Additional metabolic phenotyping was performed on the isolate using the Analytic Profile Index (API) 20E system (bioMérieux, Inc.). To differentiate between *E. coli* and *Shigella*, isolates were inoculated into Motility Media with Tetrazolium growth Indicator (Hardy Diagnostics). Isolates were also cultured on Xylose‐Lysine‐Deoxycholate (XLD) Agar, specifically to confirm the isolate's inability to metabolize Xylose (Silva et al., 1980).

### 2.2 Antibiotic resistance profile analysis

Testing via the disk diffusion method was conducted to determine the susceptibility of EcoFA807‐17 to selected members of classes of antimicrobial agents including the beta‐lactam, macrolide, fluoroquinolone, aminoglycoside, sulfonamide, tetracycline, and chloramphenicol families.

### 2.3 Species identification using multi‐locus sequence analysis

Multi‐locus sequence analysis (MLSA) was performed as part of the initial species identification process. Total genomic DNA (gDNA) was isolated from bacterial colonies using a Qiagen DNeasy Blood and Tissue Kit (Qiagen). Purified extracted genomic DNA (gDNA) was quantified for concentration with a DeNovix DS‐11 series Spectrophotometer (DeNovix Inc.). One nanogram (1 ng) of gDNA was used for each Polymerase Chain Reaction (PCR) assay. Bacterial household target genes included 16S rRNA (*rrn*), β subunit of the RNA Polymerase (*rpoB*), and DNA gyrase subunit B (*gyrB*). Gene‐specific PCR primers, developed for routine use in our group and proven against *E. coli* ATCC strain 10536, and the thermocycler parameters for these targets are listed in Table 1. PCR amplicons were purified using the Qiaquick PCR Purification Kit (Qiagen, Co.) and submitted for Sanger sequencing using the same primers (Table 1) (Genewiz, Inc.), and the sequences were analyzed using the CLC Main Workbench software (version 8.1, Qiagen Bioinformatics, Inc.). Sequence alignment and identification (Table 4) were performed using the Basic Local Alignment Search Tool (BLAST; https://blast.ncbi.nlm.nih.gov/Blast.cgi).

**Table 1:** MLSA PCR primers and thermocycler parameters

Target gene	Forward	Reverse
**16S rRNA**	5′‐CGGACGGGTGAGTAATGTCT‐3′	5′‐TCAACAACCGAGCTGACGAC‐3′
95°C for 1 min followed by 35 cycles of 95°C for 30 s, 56°C for 30 s, and 72°C for 30 s, and a final extension		
extension step of 72 C for 5 min		
***rpoB***	5′‐AGACCGTTTCACCATCC‐3′	5′‐ACCCTTGTTACGTGACGAC‐3′
94°C for 1 min followed by 35 cycles of 94°C for 30 s, 55°C for 30 s, and 72°C for 30 s, and a final extension		
step of 72 C for 5 min		
***gyrB***	5′‐GCGTAACCCGGGTATGTA‐3′	5′‐CCGTCGACGTCCGCATCGGTCAT‐3′
94°C for 1 min followed by 35 cycles of 94°C for 30 s, 60°C for 30 s, and 72°C for 30 s, and a final extension		
step of 72 C for 5 min		
***yybW*** **(region 401‐611)**	5′‐TGATTGGCAAAATCTGGCCG‐3′	5′‐GAAATCGCCCAAATCGCCAT‐3′
95°C for 3 min followed by 40 cycles of 95°C for 15 s, 62°C for 30 s, and 72°C for 30 s.		
Melt Curve 65.0°C to 95.0°C: Increment 0.5°C, 0:05 Plate Read		

Abbreviations: MLSA, multi‐locus sequence analysis; PCR, polymerase chain reaction. yybW primer sequences and PCR cycle parameters from Walker et al. (2017).

### 2.4 Amplification of the *E. coli*‐specific *ybbw* gene

PCR was performed by targeting the species‐defining *E. coli ybbW* gene as described (primer sequences and PCR parameters are listed in Table 1) (Walker et al., 2017).

### 2.5 Whole genome sequencing of isolate

Genomic DNA of EcoFA807‐17 with a concentration in excess of 4 nM was prepared via Illumina Nextera NT DNA Library Kit (Illumina). DNA sequence data for EcoFA807‐17, 5,425,374 million 151 bp paired‐end reads, was generated on the Illumina MiSeq platform in the Epigenomics and DNA Sequencing Core at NIEHS. Quality was checked with the Fastqc program (https://bioinformatics.babraham.ac.uk/projects/fastqc/) and no trimming or adaptor removal was indicated. Sequence assemblies and genome annotation were done with Patric 3.5.30 (https://www.patricbrc.org/; (Wattam et al., 2017). Assembly with this web‐based toolkit is done with the SPAdes assembler (Bankevich, Nurk, et al., 2012) and resulted in an assembly of 259 contigs. The genome size is 4,964,044 bp and the genome coverage is 330X. Annotation is based on the RASTtk annotation pipeline (Brettin et al., 2015). The annotation advantage of using Patric is that it contains seven databases related to proteins involved in bacterial pathogenesis, virulence, antibiotic resistance, and drug targets. Based on our comparison to prokka (Seemann, 2014), a standard prokaryotic annotation tool, Patric provides a much more accurate annotation of draft prokaryotic genomes and allowed us to identify the *ttr* operon.

### 2.6 Analysis of genes involved in tetrathionate respiration

Whole genome sequencing identified genes involved in tetrathionate respiration within EcoFA807‐17. To confirm their presence, we first performed PCR analysis utilizing the assay previously reported to detect the Salmonella‐specific *ttr* locus (Malorny et al., 2004). In addition, we performed PCR amplification of the individual *ttr* genes (A, B, C, R, and S) using PCR primers designed (Table 2) from the *ttrRSBCA* sequences computationally described in the annotated whole genome sequence data. To confirm the computational prediction of existing *ttr* genes, partial sequence amplicons of all five genes were successfully generated via PCR (Figure 3) and submitted for sequencing (Genewiz, Inc.), then examined for confirmation of protein function/identification and taxonomic data via the NCBI BLASTX and BLASTN Bioinformatics search tools, respectively.

**Table 2:** *TTR* operon primers and thermocycler parameters

Gene	Forward	Reverse
*ttrA*	5′‐ACAGCCCGCTACGTATTCTG‐3′	5′‐GCGGGAGTAGATAAACGCCA‐3′
*ttrB*	5′‐CGAAGGCTCACAACAGCATC‐3′	5′‐CACGTCCCATCAATGGGGTA‐3′
*ttrC*	5′‐GTTACCCTGGGCCGTACAAT‐3′	5′‐GTAACGTCCAGCGCATTAGC‐3′
*ttrR*	5′‐GGCCTGTGCGTTTTTATTGGA‐3′	5′‐TTTTGCTACCAGATGCGCCA‐3′
*ttrS*	5′‐TGTTTAACCAGTACGCCGCT‐3′	5′‐ATGGCCAGTCCTAGTCCCAT‐3′

*Note*: The thermal cycling parameters were 95°C for 3 min followed by 35 cycles of 95°C for 15 s for denaturation, 60/62/64°C (60°C ‐ *ttrA*, *ttrC*, *ttrS*, 62C ‐ *ttrB*, 64C‐ *ttrR*) for 30 s for the annealing step, and 72°C for 1 min for extension. A final extension step of 72°C for 5 min was included.

During Polymerase Chain Reaction (PCR) analysis of genes involved in tetrathionate respiration, *E. coli* (ATCC 10536) was utilized as a *ttr*(−) negative control. *Salmonella enterica* (ATCC 13076) and *Citrobacter freundii* (ATCC 8090) also served as positive controls as both possess a complete *ttr* operon.

### 2.7 Growth of EcoFA807‐17 in tetrathionate broth

Cultures of EcoFA807‐17 and *Salmonella enterica*, *Citrobacter freundii*, and *ttr*(−) *E. coli* (ATCC 10536) were grown for at least 24 h at 37°C on MacConkey agar. Individual colonies of all species were picked via 1 μl disposable loops to inoculate 16 × 150 ml screw‐top tubes containing 10 ml of tetrathionate broth (Hardy Diagnostics). One set of tubes containing all four species was amended with the addition of 200 μl of iodine‐iodide solution (Hardy Diagnostics) just before inoculation. A second corresponding set of tubes was inoculated with one of all four species without an amendment of the iodine‐iodide solution. All sets were then placed in Gas‐Pak sealed jars with CO_2_ generator packs (Becton Dickinson) to establish an anaerobic growth environment and incubated at 37°C. After 8 h, all tubes of both sets were removed and 10 μl of media from each tube was deposited on MacConkey agar plates, streaked for isolation, and placed in an aerobic incubator for an additional 8 h at 37°C.

## 3 RESULTS

### 3.1 Biochemical phenotype and antibiotic resistance profiles of EcoFA807‐17

Biochemical profile data of EcoFA807‐17 for purposes of taxonomic determination is recorded in Table 3 (with commensal reference strain *E. coli* ATCC 10536) were derived in part from the Analytic Profile Index (API) 20E system (see Methods and Materials) and from differential media including TSI slant agar for sugar utilization and H_2_S production, XLD agar for Xylose utilization, and semi‐solid Motility media.

**Table 3:** Biochemical phenotype of EcoFA807‐17 compared with *E. coli* (ATCC 10536)

Biochemical test	EcoFA807‐17	*E. coli* ATCC 10536
ONPG	−	+
Arginine decarboxylation	−	+
Lysine decarboxylation	−	+
Ornithine decarboxylation	−	+
Citrate utilization	−	−
Hydrogen sulfide production	−	−
Urease	−	−
Tryptophan deaminase	−	−
Indole	+	+
Vogues–Proskauer	−	−
Gelatinase	−	−
Glucose	+	+
Mannose	+	+
Inositol	−	−
Sorbitol	+	+
Rhamnose	+	+
Sucrose	−	+
Melibiose	+	+
Amygdalin	−	−
Arabinose	+	+
Lactose	−	+
Xylose	−	+
Triple sugar iron test	Alkaline/acid/slight gas	Acid/acid/gas
Motility	Motile	Motile

Apart from tetrathionate respiration, the metabolic profile of EcoFA807‐17 differs from commensal strains of *E. coli* in several ways. It does not decarboxylate lysine or ornithine which are known virulence factors among *Escherichia/Shigella* genomospecies (Casalino et al., 2003; Gomig et al., 2015; Maurelli et al., 1998), although it demonstrates Indole production, a trait long known to be typical of *E. coli* but much less common with *Shigella* (Rezwan et al., 2004) and certain other members of Enterobacteriaceae. Its sugar utilization resembles *Shigella* in being unable to metabolize lactose, sucrose, and xylose (Taylor, 1965). However, EcoFA807‐17 differs from *Shigella* spp. in being robustly motile (Beld & Reubsaet, 2012). In contrast to many pathogens in which wide‐spectrum antibiotic resistance is frequently characteristic, EcoFA807‐17 was found to be generally susceptible to a broad array of antibiotic agents (Table 4).

**Table 4:** EcoFA807‐17 antibiotic analysis

Antibiotic/antibiotic class	Diameters of inhibition zones (mm)	MIC results
Ampicillin 10 µg/beta‐lactams	24	Susceptible
Azithromycin 15 µg/macrolides	30	Susceptible
Aztreonam 30 µg/beta‐lactams	39	Susceptible
Cephalothin 30 µg/beta‐lactams	27	Susceptible
Ciprofloxacin 5 µg/fluoroquinolones	46	Susceptible
Chloramphenicol 30 µg/chloramphenicol	34	Susceptible
Gentamicin 10 µg/aminoglycosides	28	Susceptible
Kanamycin 30 µg/aminoglycosides	22	Susceptible
Meropenem 10 µg/beta‐lactams	37	Susceptible
Sulfamethoxazole/trimethoprim 1.25 µg/sulfonamides	32	Susceptible
Tetracycline 30 µg/tetracyclines	25	Susceptible
Tigecycline 15 µg/glycylcyclines (derived from tetracyclines)	29	Susceptible

*Note*: Used Mueller–Hinton agar.

Abbreviation: MIC, minimum inhibitory concentration.

**Figure 2:**
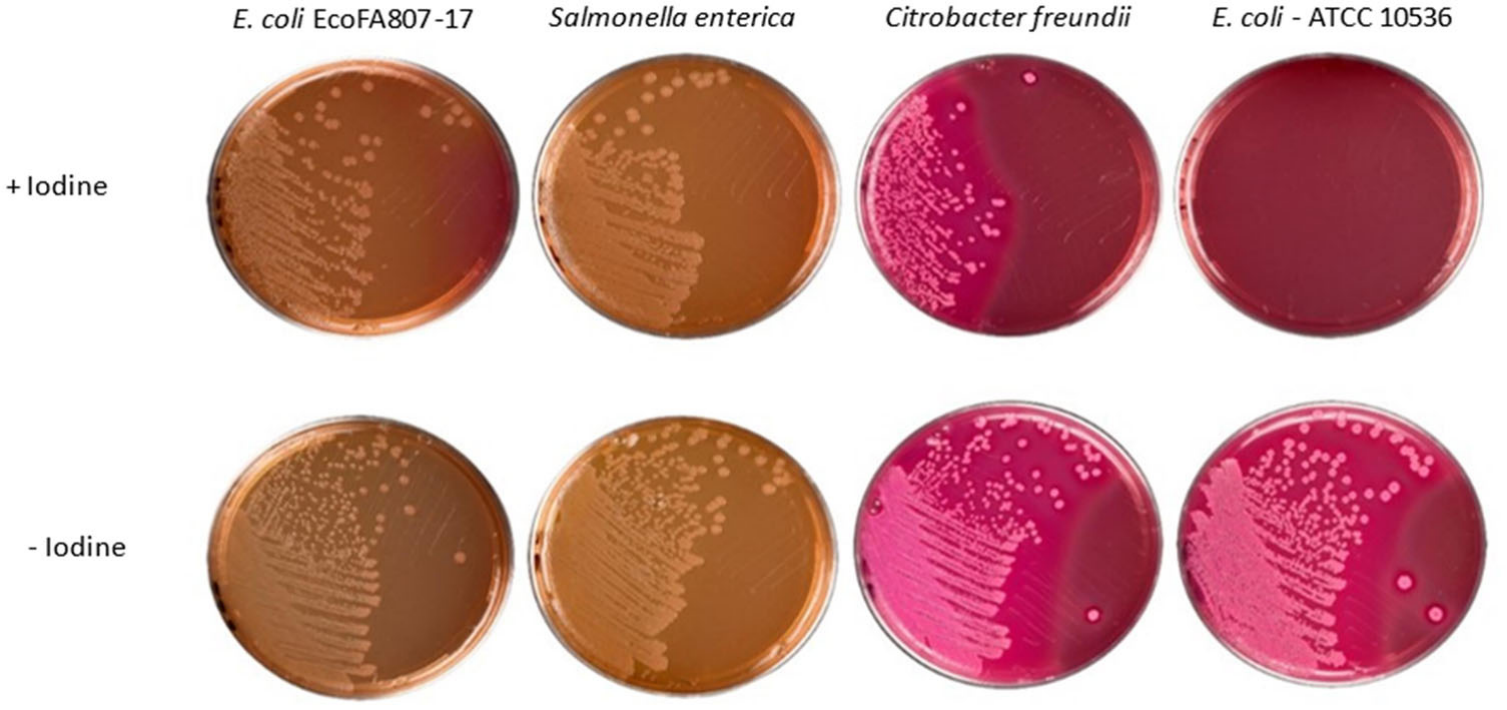
Bacterial growth on MacConkey agar after 8 h in tetrathionate with and without iodine.

When cultured under anaerobic conditions in (Iodine‐Iodide) activated tetrathionate broth to simulate an environment of inflammation within the gut, EcoFA807‐17 robustly demonstrated the biological activity of its suite of *ttr* genes with growth similar to the positive control *Salmonella* and *Citrobacter* strains. Conversely, the *ttr*(−) strain *E. coli* remained inhibited and failed to grow at the same rate as EcoFA817‐17 and the other *ttr*(+) species when transferred to MacConkey agar (Figure 2). This result was further emphasized with the successful culture of *ttr*(−) *E. coli* on MacConkey agar derived from tetrathionate broth that had not been amended with the iodine‐iodide solution as seen in Figure 2.

### 3.2 Multi‐locus sequence analysis of EcoFA807‐17

The housekeeping gene targets in Table 5 were examined via NCBI BLAST for sequence similarity, confirming EcoFA807‐17 as a member of the *E. coli/Shigella* genomospecies.

**Table 5:** Leading NCBI BLAST matches to EcoFA807‐17

Target gene	Leading NCBI BLAST matches to EcoFA807‐17	Nucleotide identity (%)
16S (*rrn*)	*S. sonnei* strain ECSW + 14	99.77
	*E. coli* strain K‐12, sub‐strain MG1655	99.77
	*E. coli* O157:H7 strain Sakai	99.77
*rpoB*	*E. coli* O157:H7 strain Sakai	99.70
	*Shigella sonnei* strain ECSW + 14	99.26
	*E. coli* strain K‐12, sub‐strain MG1655	99.26
*gyrB*	*Shigella sonnei* strain ECSW + 14	99.05
	*E. coli* strain K‐12, sub‐strain MG1655	99.05
	*Shigella dysenteriae* strain BCW_4872	98.84

To further differentiate between *E. coli* and *Shigella*, the presence of a *ybbW* (allantoin permease) allele was affirmed by rtPCR assay and by computational prediction derived from whole genome sequencing.

### 3.3 Sequencing analysis of *ttr* elements

**Figure 3:**
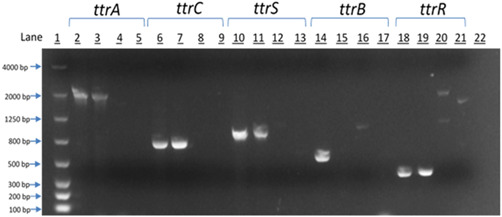
The PCR amplicons of the various *ttr* locus elements were resolved on a 2.0% agarose gel. The lanes are as follows: 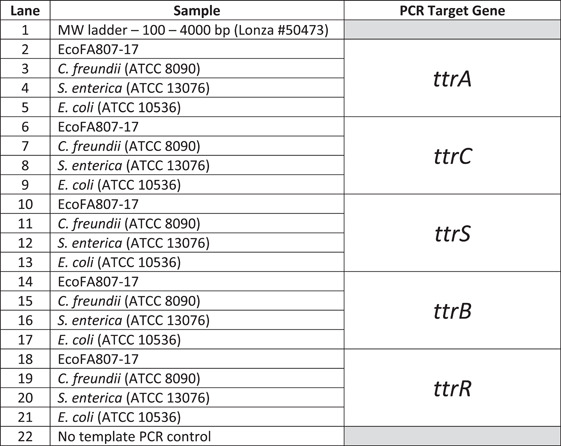 *Note*: Trace bands in Lanes 16, 20, and 21 were subjected to additional PCR and determined via sequencing and BLAST analysis to be nonspecific amplification.

To confirm the computational prediction of existing *ttr* genes, partial amplicons of all five genes were created via PCR and submitted for sequencing (Genewiz, Inc.), then examined for confirmation of protein identification and taxonomic data via the NCBI BLASTX and BLASTN Bioinformatics search tools, respectively. Despite the significant divergence in nucleotide identity between the EcoFA807‐17 and *C. freundii* homologs, we were consistently successful in amplifying most of the *ttr* elements of both species with primers specifically targeting EcoFA807‐17 *ttr* homologs, except for the *ttrB* structural gene of *C. freundii* (Figure 3, Lane 15), using stringent annealing temperatures consistent with primer melting temperatures (Tm). By contrast, we were unable to amplify *Salmonella ttr* homologs, nor did we detect the presence of *ttr* elements in EcoFA807‐17 via a Salmonella‐specific PCR assay (Malorny et al., 2004), thus providing additional support for the possibility that EcoFA807‐17 has acquired its *ttr* elements from a *Citrobacter* lineage.

#### 3.3.1 Distribution of the *ttr* operon in *E. coli* strains available at NCBI

All 20833 *E. coli* genomes listed in the *E. coli* Genome Assembly and Annotation Report (https://www.ncbi.nlm.nih.gov/genome/genomes/167/) were downloaded on 22 September 2020, and searched for those potentially containing genes in the *ttr* operon using the proteins of the *ttr* operon of EcoFA807‐17 as queries. The number of strains containing each of the five genes of this operon and the subset of that containing all five genes of the *ttr* operon are listed in Table 6. Only approximately 0.7% of these genomes contained all five genes comprising the *ttr* operon, whereas the *lacZ* gene common to coliform bacteria (Molina et al., 2015), and the *ybbW* gene, diagnostic of *E. coli*, were present in 97.8 and 96.3 of this *E. coli* genome data set, respectively.

**Table 6:** Incidence of *ttr* genes relative to common household genes (*ybbW, lacZ*) in *E. coli* genomes in NCBI

Gene	Genomes containing gene	Percent of total genomes with gene
*ttrR*	166	0.79
*ttrS*	166	0.79
*ttrB*	165	0.79
*ttrC*	167	0.80
*ttrA*	163	0.78
*ttr operon*	146	0.70
*lacZ*	20,380	97.80
*ybbW*	20,075	96.30

To confirm that these genes were present in a true operon configuration, EcoFA807‐17 and a sample of 27 complete *E. coli* genomes of those strains in Table 7 predicted to have a complete *ttr* operon were re‐annotated using the Patric annotation pipeline described in Methods. Where the assembly annotation indicated a plasmid localization of the *ttr* operon is also noted in Table 7.

**Table 7:** *E. coli* genomes containing complete *ttr* operons

Strain possessing complete ttr operon	Date submitted to NCBI	Accession	Operon locus
2.4168	2‐May‐12	GCA_000194555.2	Chromosome
541‐15	23‐May‐12	GCA_000264115.1	Chromosome
KTE111	5‐Apr‐13	GCA_000351865.1	Chromosome
KTE40	4‐Jun‐13	GCA_000408025.1	Chromosome
KTE64	4‐Jun‐13	GCA_000408365.1	Chromosome
UMEA 3052‐1	29‐Aug‐13	GCA_000460035.1	Chromosome
UMEA 3889‐1	29‐Aug‐13	GCA_000461755.1	Chromosome
UMEA 3148‐1	1‐Nov‐13	GCA_000495075.1	Chromosome
D6‐117.29	11‐May‐14	GCA_000723325.1	Chromosome
2‐156‐04_S4_C2	11‐Jun‐14	GCA_000704025.1	Chromosome
2‐474‐04_S1_C1	11‐Jun‐14	GCA_000704565.1	Chromosome
2‐474‐04_S1_C2	11‐Jun‐14	GCA_000704645.1	Chromosome
CVM N41498PS	7‐Dec‐14	GCA_000797915.1	Chromosome
8.0569	18‐Mar‐15	GCA_000305355.1	Chromosome
YE16	7‐Aug‐15	CXWW01000045.1	Chromosome
YE19	7‐Aug‐15	CXYJ01000044.1	Chromosome
AW1.7 1161	9‐Oct‐15	GCA_001309455.1	Chromosome
AW1.7‐delta‐pHR1 996	9‐Oct‐15	GCA_001309475.1	Chromosome
GM16‐6	9‐Oct‐15	GCA_001309555.1	Chromosome
RU1	26‐Oct‐15	GCA_001413005.1	Chromosome
RU1 MA1	26‐Oct‐15	GCA_001413355.1	Chromosome
RU1 MA3	26‐Oct‐15	GCA_001413395.1	Chromosome
RU1 MA4	26‐Oct‐15	GCA_001413415.1	Chromosome
RU1 MA5	26‐Oct‐15	GCA_001413425.1	Chromosome
RU1 MA6	26‐Oct‐15	GCA_001412895.1	Chromosome
RU1 MA7	26‐Oct‐15	GCA_001413455.1	Chromosome
RU1 MA8	26‐Oct‐15	GCA_001412915.1	Chromosome
RU1 MA9	26‐Oct‐15	GCA_001413475.1	Chromosome
RU1 MA10	26‐Oct‐15	GCA_001413485.1	Chromosome
RU1 MA11	26‐Oct‐15	GCA_001413515.1	Chromosome
RU1 MA12	26‐Oct‐15	GCA_001413535.1	Chromosome
RU1 MA12	26‐Oct‐15	GCA_001413535.1	Chromosome
RU1 LB1	26‐Oct‐15	GCA_001412925.1	Chromosome
RU1 LB2	26‐Oct‐15	GCA_001412955.1	Chromosome
RU1 LB3	26‐Oct‐15	GCA_001413555.1	Chromosome
RU1 LB4	26‐Oct‐15	GCA_001413565.1	Chromosome
RU1 LB5	26‐Oct‐15	GCA_001412975.1	Chromosome
RU1 LB6	26‐Oct‐15	GCA_001413595.1	Chromosome
RU1 LB7	26‐Oct‐15	GCA_001413605.1	Chromosome
RU1 LB9	26‐Oct‐15	GCA_001412995.1	Chromosome
RU1 LB10	26‐Oct‐15	GCA_001413645.1	Chromosome
RU1 LB11	26‐Oct‐15	GCA_001413675.1	Chromosome
RU1 LB12	26‐Oct‐15	GCA_001413685.1	Chromosome
RU1 BHI2	26‐Oct‐15	GCA_001413735.1	Chromosome
RU1 BHI3	26‐Oct‐15	GCA_001413745.1	Chromosome
RU1 BHI4	26‐Oct‐15	GCA_001413755.1	Chromosome
RU1 BHI5	26‐Oct‐15	GCA_001413795.1	Chromosome
RU1 BHI7	26‐Oct‐15	GCA_001413875.1	Chromosome
RU1 BHI9	26‐Oct‐15	GCA_001413885.1	Chromosome
RU1 BHI11	26‐Oct‐15	GCA_001413905.1	Chromosome
RU1 BHI12	26‐Oct‐15	GCA_001413825.1	Chromosome
AZ71	4‐Jan‐16	GCA_001484375.1	Chromosome
G138	26‐Feb‐16	GCA_001575835.1	Chromosome
G150	26‐Feb‐16	GCA_001575865.1	Chromosome
G184	26‐Feb‐16	GCA_001575945.1	Chromosome
G186	26‐Feb‐16	GCA_001576295.1	Chromosome
G228	26‐Feb‐16	GCA_001575705.1	Chromosome
G239	26‐Feb‐16	GCA_001575695.1	Chromosome
G240	26‐Feb‐16	GCA_001575635.1	Chromosome
G3	26‐Feb‐16	GCA_009823265.1	Chromosome
G35	26‐Feb‐16	GCA_001576385.1	Chromosome
AF7945	29‐Sep‐16	GCA_001749075.1	Chromosome
639	8‐Dec‐16	GCA_001893305.1	Chromosome
643	8‐Dec‐16	GCA_001893215.1	Chromosome
491	8‐Dec‐16	GCA_001893375.1	Chromosome
552	8‐Dec‐16	GCA_001893225.1	Chromosome
584	8‐Dec‐16	GCA_001894315.1	Chromosome
602	8‐Dec‐16	GCA_001893995.1	Chromosome
647	8‐Dec‐16	GCA_001894425.1	Chromosome
684	8‐Dec‐16	GCA_001893765.1	Chromosome
H15	12‐Dec‐16	GCA_001901005.1	Plasmid
299.h	1‐Sep‐17	GCA_002284735.1	Chromosome
F1_405E	6‐Sep‐17	GCA_900195635.1	Chromosome
MOD1‐EC3803	5‐Oct‐17	GCA_002516065.1	Chromosome
MOD1‐EC3800	5‐Oct‐17	GCA_002516125.1	Chromosome
MOD1‐EC6602	6‐Oct‐17	GCA_002467595.1	Chromosome
MOD1‐EC5157	6‐Oct‐17	GCA_002516885.1	Chromosome
MOD1‐EC6855	11‐Oct‐17	GCA_002520525.1	Chromosome
MOD1‐EC6132	13‐Oct‐17	GCA_002537315.1	Chromosome
MOD1‐EC6011	13‐Oct‐17	GCA_002542995.1	Chromosome
MOD1‐EC6007	13‐Oct‐17	GCA_002538315.1	Chromosome
MOD1‐EC5984	13‐Oct‐17	GCA_002543795.1	Chromosome
YH17167	25‐Feb‐18	GCA_002941405.1	Chromosome
EBJ001	3‐Mar‐18	GCA_002967885.1	Chromosome
B29595	8‐Mar‐18	GCA_003007795.1	Chromosome
KG‐3	11‐Mar‐18	GCA_002993705.1	Chromosome
13561‐5	31‐Mar‐18	GCA_003027475.1	Chromosome
KG‐18	10‐Apr‐18	GCA_003048005.1	Chromosome
Win2012_WWKa_NEU_31	21‐May‐18	GCA_003145195.1	Chromosome
Sum2013_WWKa_OUT_2	21‐May‐18	GCA_003145395.1	Chromosome
Spr2013_WWKa_ALT_27	21‐May‐18	GCA_003145945.1	Chromosome
Spr2012_WWKa_NEU_74	21‐May‐18	GCA_003146135.1	Chromosome
TUM15671	9‐Jun‐18	GCA_003227915.1	Chromosome
VREC0596	18‐Jun‐18	GCA_900480485.1	Chromosome
VREC0525	18‐Jun‐18	GCA_900481835.1	Chromosome
VREC0631	18‐Jun‐18	GCA_900482185.1	Chromosome
A24	9‐Jul‐18	GCA_003292515.1	Chromosome
A39	9‐Jul‐18	GCA_003292875.1	Chromosome
1262	9‐Jul‐18	GCA_003293115.1	Chromosome
A48	9‐Jul‐18	GCA_003293255.1	Chromosome
1257	9‐Jul‐18	GCA_003301735.1	Chromosome
JL39	9‐Jul‐18	GCA_003291015.1	Chromosome
GER_MD01_1509_Eco_058	15‐Jul‐18	GCA_003322075.1	Chromosome
GER_MD11_1505_Eco_029	15‐Jul‐18	GCA_003322395.1	Chromosome
GER_MD11_1505_Eco_027	15‐Jul‐18	GCA_003322415.1	Chromosome
GER_MD11_1505_Eco_023	15‐Jul‐18	GCA_003322465.1	Chromosome
KL53	20‐Jul‐18	GCA_002494365.2	Plasmid
NCTC11130	30‐Jul‐18	GCA_900449175.1	Chromosome
NCTC13384	31‐Jul‐18	GCA_900448435.1	Chromosome
NCTC11106	31‐Jul‐18	GCA_900448865.1	Chromosome
HPC‐781c	20‐Sep‐18	GCA_003583685.1	Chromosome
PN112	26‐Nov‐18	GCA_003830335.1	Chromosome
PN91	26‐Nov‐18	GCA_003830385.1	Chromosome
GBGD39	3‐Dec‐18	GCA_003858925.1	Chromosome
NCTC11129	19‐Dec‐18	GCA_900636075.1	Chromosome
49_rectal	21‐Jan‐19	GCA_004100615.1	Chromosome
RS571	25‐Jan‐19	GCA_004114395.1	Plasmid
071H1	6‐Feb‐19	GCA_004173925.1	Chromosome
93‐I92‐A	14‐Mar‐19	GCA_005402105.1	Chromosome
URMC_46	2‐Apr‐19	GCA_004567595.1	Chromosome
KCJK7052	9‐Apr‐19	GCA_004767325.1	Chromosome
BE565	16‐Apr‐19	GCA_005381805.1	Chromosome
JML241	16‐Apr‐19	GCA_005388945.1	Chromosome
EC‐129	6‐May‐19	GCA_005156265.1	Plasmid
SCEC020023	19‐Aug‐19	GCA_002850675.5	Plasmid
CD64_9	20‐Aug‐19	GCA_008040635.1	Chromosome
1_53_1	20‐Aug‐19	GCA_008041255.1	Chromosome
1_52_13	20‐Aug‐19	GCA_008041305.1	Chromosome
P042A	27‐Aug‐19	GCA_008120445.1	Chromosome
C27A	18‐Dec‐19	GCA_009762475.1	Plasmid
SH9c	22‐Dec‐19	GCA_009789905.1	Chromosome
IH27c	22‐Dec‐19	GCA_009791155.1	Chromosome
IH8c	22‐Dec‐19	GCA_009791245.1	Chromosome
IH3c	22‐Dec‐19	GCA_009791265.1	Chromosome
G39	31‐Dec‐19	GCA_001576155.1	Chromosome
8374wF12	14‐Jan‐20	GCA_009882595.1	Chromosome
ATCC 11231	10‐Feb‐20	GCA_010374855.1	Chromosome
ATCC 11229	10‐Feb‐20	GCA_010374945.1	Chromosome
EC8	25‐Feb‐20	GCA_011008605.1	Chromosome
CAP45	26‐Feb‐20	GCA_011028105.1	Chromosome
CAP36	26‐Feb‐20	GCA_011032125.1	Chromosome
169757	29‐Feb‐20	GCA_011043615.1	Plasmid
Fec 10	23‐Jun‐20	WP_001076427.1	Plasmid
HUM‐546	13‐Jul‐20	GCA_013404135.1	Chromosome
G43	17‐Jul‐20	GCA_001576495.1	Chromosome
GCPRC7	28‐Jul‐20	GCA_013850805.1	Chromosome

A similar strategy was used to search available NCBI *Shigella* genomes, as of 5 May 2020. One *S. sonnei* strain was found to possess the *ttr* operon, as were just three other additional Shigella strains including one strain of *S. flexneri* and two *Shigella* strains of undetermined species. No *S. dysenteriae* or *S. boydii* genomes were found to contain a *ttr* operon.

*Shigella* strains containing a complete *ttr* operon are listed in Table 8. *Shigella* genomes were formatted into a BLAST database and searched for the five protein queries from the *ttr* operon from EcoFA807‐17 by BLAST. Dates of isolation (where available) and NCBI accession numbers are given in columns 3 and 4 of Table 8. One additional strain, *Shigella spp*. FC1967 (GCA_001743005.1) possessed four of the five genes found in the *ttr* operon.

**Table 8:** Shigella genomes containing the ttr operon

Species	*Ttr(+) strain*	Accession	Year submitted	*ybbW and ttr*(+)	*ybbW* (+) genomes *
*S. sonnei*	ESCW+10	GCA_002248745.1	2017	yes	30
*S. flexneri*	1235‐66	GCA_000268065.1	2012	no	11
*Shigella* spp.	FC1655	GCA_001742985.1	2016	no	NA
*Shigella* spp.	FC130	GCA_001722135.1	2016	no	NA
*Shigella* spp.	FC1967	GCA_001743005.1	2016	no	NA

*Note*: (*) number of *ybbW* (+) Shigella species genomes deposited in GenBank.

## 4 DISCUSSION

### 4.1 Taxonomic determination of species

As *E. coli* had not previously been reported to possess tetrathionate reductase genes (Palumbo & Alford, 1970; Price‐Carter et al., 2001; Roth, 2017), confirmation of the taxonomic classification of EcoFA807‐17 as *E. coli* was essential. This strain presents a conflicting and atypical biochemical identity profile, particularly confounding differentiation between *E. coli* and *Shigella*. EcoFA807‐17 resembles *Shigella* in some respects rather than *E. coli*, most strikingly by its inability to ferment xylose (Altwegg et al., 1996; de Boer, 1998; Taylor, 1965). Further complicating differentiation between *E. coli* and the obligate pathogen *Shigella*, the close phylogenetic relationship between the two organisms thwarts the use of household genes typically used in multi‐locus sequence analysis for identification at the species level. Those MLSA targets used for the initial genetic examination include *rrn (*ribosomal RNA or 16S*), gyrB, and rpoB*. The sequencing and attempted species identification via these genes deserve further comment. Long considered the “gold standard” for taxonomic classification (Case et al., 2007) of bacterial species, the *rrn (*16S*)* gene is effective for taxonomic determination at the genus level and sometimes at the species level as well, but is unable to decisively discriminate between *E. coli* and *Shigella (*Christensen et al., 1998; Devanga Ragupathi et al., 2018), although its employment was initially instrumental in confirming that EcoFA807‐17 was not *Salmonella*. This was not surprising given that *Escherichia* and *Shigella*, while deemed separate taxa for purposes of clinical distinction (Farmer et al., 1985; Rezwan et al., 2004), are genetically the same species (Beld & Reubsaet, 2012; Fukushima et al., 2002). They are therefore too closely related to be reliably differentiated by the highly conserved and frequently multi‐copy ribosomal RNA genes (Devanga Ragupathi et al., 2018). Similarly, the *gyrB* and *rpoB* household genes which are generally more discriminatory than *rrn* (16S) at the species and even sub‐species level, were determined nonetheless to be insufficiently useful to differentiate between *E. coli* and *Shigella* as demonstrated by inconclusive results (Table 5) obtained from NCBI data of the housekeeping gene sequences submitted. By extension, they are also deemed wholly unsuitable for serovar/biovar determination (Adékambi et al., 2009).

Accordingly, a real‐time PCR assay described by Walker et al., (Walker et al., 2017) was utilized to enable a decisive confirmation of EcoFA807‐17 as *E. coli*. The principle underlying this method is that the *ybbW* gene, which codes for allantoin permease, has been regarded as universally inclusive and exclusive to *E. coli* (but not *Shigella*) and subsequent testing confirmed that EcoFA807‐17 is *ybbW* (+). We observe, however, that some data from this study suggests that *ybbW* may not be as exclusive to *E. coli* as originally thought. Specifically, we note that a number of strains of *S. sonnei* and S. *flexneri* found within NCBI data (Table 8, right column) appear to possess the *ybbW* gene, including *ttr(+) S. sonnei* strain ECSW + 10 (GCA_002248745.1). In the case of the latter, while conceivable that ECSW + 10 is an *E. coli* (EIEC) strain originally misclassified as *S. sonnei*, this is a less likely explanation as further examination of the genome of *S. sonnei* strain ECSW + 10 reveals the predicted presence of a pINV type B invasion plasmid. The latter is characteristic of *S. sonnei* while closely related EIEC strains and certain other Shigellae tend to possess pINV Type A invasion plasmids (Lan et al., 2001, 2004). The unexpected presence of *ybbW* as well as a *ttr* operon within *S. sonnei* strain ECSW + 10 serves to further emphasize the dynamic mutability of the *E. coli/Shigella* pan‐genome. Nevertheless, the presence of the *ybbW* gene is observed to be uncommon among the several thousand *Shigella* genomes deposited at NCBI. For that reason and because the Shigellae universally possess pINV invasion plasmids and lack motility (Beld & Reubsaet, 2012), we conclude that EcoFA807‐17, which lacks the former and is highly motile, is properly classified as a strain of *E. coli*.

### 4.2 Characterization of the *ttr* operon of EcoFA807‐17

Initially (in 2018), the *ttr* operon sequences of EcoFA807‐17 were determined via NCBI BLASTN to be most closely related to *Citrobacter freundii* homologs with shared nucleotide identity ranging from 85% to 90% as described from NCBI data. Reinforcing this data, we found that our *ttr* PCR primers, designed from sequences predicted of EcoFA807‐17, not only successfully PCR‐amplified and confirmed the computationally indicated existence of these genes, but also PCR‐amplified four out of the five counterparts found in *C. freundii* (see Figure 3), despite the significant degree of deviation in nucleotide identity of the latter species. Recently, a follow‐up query to NCBI revealed new sequences had become available, and we have determined that all *ttr* elements in EcoFA807‐17 are much more closely related to homologs found within *Citrobacter amalonaticus* and to a slightly lesser extent, a novel species *Citrobacter portucalensis* (Ribeiro et al., 2017), in both cases sharing approximately 99% identity with EcoFA807‐17. Of special note with respect to *C. portucalensis*, due to its recent taxonomic classification, only a few studies relevant to this species have been published and none have reported its possession of *ttr* genetic elements. As for the uncommon presence of the *ttr* operon in *E. coli*, regarding its origin and nearly perfect nucleotide identity with certain homologs within *Citrobacter*, it is most likely the result of horizontal transfer from a related species. The EcoFA807‐17 genome was examined for plasmid content using three tools: plasmidSpades, Recycler (https://github.com/Shamir-Lab/Recycler#bam-prep), and PlasmidSeeker (https://github.com/bioinfo-ut/PlasmidSeeker). Two plasmids predicted by all three tools were considered candidate plasmids. Based on BLAST analysis, the *ttr* operon locus is not on either of these two plasmids and thus is likely chromosomal. This is consistent with the fact that of the *ttr(+)* strains listed in Table 7, relatively few appear to possess plasmid‐borne *ttr* elements. To address the question of whether *ttr* elements within EcoFA807‐17 were biologically active, the strain was cultured anaerobically in tetrathionate broth as previously described. Simulation of the in vivo infection and inflammation process is achieved with the addition of the Iodine‐iodide solution to the broth. Metabolism of tetrathionate by bacteria imparts a growth advantage under anaerobic conditions and requires the possession of a fully functional *ttr* operon. Tetrathionate broth has been commonly used for the cultivation of Salmonella specifically because it inhibits growing cells of many *ttr*(−) Gram‐negative species, and in particular, coliforms such as *E. coli*. As with the *Salmonella* and *Citrobacter* positive control strains, EcoFA807‐17 was easily cultured in tetrathionate broth, whereas the *ttr* negative control strain of *E. coli* remained inhibited, except in tetrathionate broth that had not been oxidized via the addition of iodine‐iodide solution.

### 4.3 Potential origins of the acquisition of ttr operon

We approached the question of origin for the type of *ttr* operon now established in the *E. coli*/Shigella pan‐genome with an examination of individual *ttr* gene homology within all known *ttr(+)* strains among *E. coli* (146 in total) and an assessment of the syntenic conservation of genetic elements immediately upstream and downstream of the *ttr* operon chromosomal loci, using EcoFA807‐17 and twenty‐seven randomly chosen *ttr(+) E. coli* strains from Table 7. In choosing the *ttrA* structural gene as representative for the operon, we ascertained that EcoFa807‐17 shared 100% nucleotide identity with approximately 51% of strains of *ttr(+) E. coli* (74/146) and all of the very few known *ttr(+)* Shigella strains (total of 5). 43% of *ttr(+)* strains (63/146) shared between 99% and 100% nucleotide identity with EcoFA807‐17. The remaining 6% of *ttr(+)* strains possessed *ttr* homologs considerably below the threshold of 99% nucleotide shared identity. This is consistent with the fact that this study has earlier determined that *ttr* homologs within the genus *Citrobacter* possess varying degrees of sequence conservation as demonstrated by the significant difference between *C. freundii* and *C. amalonaticus*. We then further examined the synteny of chromosomal elements in the immediate vicinity of the ttr operon among the same twenty‐eight *ttr(+) E. coli* genomes including EcoFA807‐17. A common feature of the surrounding loci revealed the frequent presence of the Methionine ABC transporter substrate binding and Fumarate hydratase genes within ~86% of the *E. coli* strains sampled, immediately adjacent upstream and downstream respectively of the operon, while every strain possessed at least one of these two flanking genes. Extending this analysis to homologs examined within *Citrobacter freundii* (ATCC 8090), *Citrobacter portucalensis* (strain FDAARGOS_617), and *Salmonella enterica* (ATCC 13076), the same association of the aforementioned adjacent genes is uniformly present in both *Citrobacter* species, whereas only the Fumarate hydratase gene is associated with the *Salmonella ttr* operon. Beyond these two operon‐flanking genes, however, the degree of conserved synteny among these 28 strains degenerated immediately thereafter. Finally, we further attempted to address the additional question of whether *Salmonella ttr* genes have perhaps breached the genome of any strain of *E. coli* or Shigella found in public databases but found that there is insufficient evidence that this has ever occurred. In consideration of these findings, one main conclusion is apparent. Variation in the content of genes in the immediate vicinity of the *ttr* operon coupled with the presence of nearby mobile genetic elements on either side (insertional sequences, transposases, and assorted others) strongly suggest that acquisition of the *ttr* operon within the *E. coli*/Shigella pan‐genome is the result of horizontal transfer that has occurred on multiple occasions, with gene homology indicating various *Citrobacter* species as the most likely point(s) of origin.

### 4.4 Significance of *ttr* operon acquisition

There is a relative paucity of literature before this century concerning tetrathionate respiration in the Enterobacteriaceae family since the phenomenon was first described by Pollock and co‐workers in the early 1940s (Kapralek, 1972). However, studies conducted in the 1970s by Kapralek and Rezbova with their work confined to the genus *Citrobacter*, revealed that tetrathionate respiration enhanced specific growth rate, raised growth yield, and enabled growth on non‐fermentable carbon sources. By 1999, Hensel and colleagues had identified and mapped the structure and functions of the operon responsible for tetrathionate metabolism in *Salmonella* (Hensel et al., 1999). Even then, the role of tetrathionate reductase as a virulence factor was not yet established as Hensel surmised that *ttr* metabolism was a survival trait utilized by *Salmonella* in the external environment rather than within eukaryotic hosts. This assumption is understandable, given that there were no known sources of tetrathionate in the mammalian host (Winter et al., 2010). Nevertheless, by 2010, Winter, Baumler, and associates had established that by process of induced gut inflammation and under anaerobic conditions, thiosulfate, which is endogenous within mammalian hosts, is oxidized to tetrathionate whereby the latter then becomes a terminal electron acceptor enabling respiration. As Bliska et al. noted, “one of the most important functions of the microbiota of the mammalian intestine is to promote resistance to colonization by pathogens” (Bliska & van der Velden, 2012). Winter et al. demonstrated that *Salmonella* and presumably other pathogens capable of tetrathionate reduction and respiration, gain a competitive growth advantage over fermentation‐dependent gut microbiota in anoxic environments (Winter et al., 2010).

### 4.5 Pathogenic potential of ttr(+) *Escherichia coli*

Given the available subject literature, studies of tetrathionate respiration as a virulence factor have generally focused on the obligate food‐borne pathogen *Salmonella enterica*. The genus *Citrobacter*, many species of which are known to possess the *ttr* operon, is closely related to *Escherichia* and it should be re‐emphasized that the *ttr* elements of *E. coli* strain EcoFA807‐17 are much more closely related to *Citrobacter* in terms of nucleotide identity and significantly more distantly related to the *Salmonella ttr* operon. Composed of thirteen species accepted by the International Committee on Systematics of Prokaryotes (Ribeiro et al., 2017), some *Citrobacter* are recognized as opportunistic pathogens such as the *ttr*(+) species *C. freundii* and *C. amalonaticus* (Lipsky et al., 1980). Nevertheless and apart from *Salmonella*, the literature is sparse concerning the role of tetrathionate respiration as a virulence factor in other bacteria (Hensel et al., 1999) including *Citrobacter*. Against this backdrop, predictions of *ttr*‐mediated virulence in EcoFA807‐17 are confined to the realm of speculation pending further study, even as this novel strain clearly possesses other genetic hallmarks suggesting pathogenicity. These potentially virulent elements include intriguing examples such as *eae* (Intimin) and *prp* (Pathogenesis‐related Protein), an enigmatic rare gene detected in only nine *E. coli* genomes obtained from NCBI. Additionally, it is interesting to note that this isolate's Type VI secretion system (T6SS) appears to be of similar lineage to those of *Shigella sonnei*, st. 53 G and EHEC O157:H7. In the case of the former, it has been recently reported that *S. sonnei* possesses a T6SS which specifically “competes against *E. coli* and *S. flexneri*, both in vitro and in vivo.” (Anderson et al., 2017) As tetrathionate respiration also serves to promote those organisms possessing that capability in interbacterial competition, EcoFA807‐17 may potentially have a formidable advantage over endogenous microbiota.

Our data (Table 7) suggests that the operon is gradually proliferating through the past decade in *Escherichia coli*, though it is present in less than 1% of genomes deposited at NCBI. In contrast, its presence in *Shigella* (Table 8) is extremely rare, having been found in just five strains among over 2000 genomes examined. In any event, it is now clear that the tetrathionate reductase operon is becoming established within the *Escherichia coli*/*Shigella* pan‐genome, with future implications yet to be determined.

## AUTHOR CONTRIBUTIONS

**Floyd G. Adsit**: Conceptualization (lead); data curation (lead); formal analysis (lead); investigation (lead); methodology (equal); writing–original draft (lead); writing–review & editing (equal). **Thomas A. Randall**: Formal analysis (equal); investigation (equal); writing–original draft (equal); writing–review & editing (equal). **Jacqueline Locklear**: Investigation (supporting); writing–review & editing (supporting). **David M. Kurtz**: Funding acquisition (lead); resources (equal); supervision (lead); writing–original draft (equal); writing – review & editing (equal).

## CONFLICT OF INTEREST

None declared.

## ETHICS STATEMENT

None required.

## Data Availability

In addition to the sequence submission to the Short Read Archive (accession number SRR14216030), this Whole Genome Shotgun project has also been deposited at DDBJ/ENA/GenBank under the accession JAGRQF000000000: The version described in this paper is version JAGRQF010000000: https://www.ncbi.nlm.nih.gov/bioproject/PRJNA721428.
